# Molecular characterization and evaluation of the emerging antibiotic-resistant *Streptococcus pyogenes* from eastern India

**DOI:** 10.1186/s12879-016-2079-9

**Published:** 2016-12-12

**Authors:** Dipanwita Ray, Somnath Saha, Sukanta Sinha, Nishith Kumar Pal, Basudev Bhattacharya

**Affiliations:** 1 Biochemistry Research Wing, Department of Biochemistry, Dr. B C Roy Post Graduate Institute of Basic Medical Education and Research (IPGME&R), 244B, A J C Bose Road, Kolkata, 700020 India; 2 Health and Family Welfare Department, Directorate of Medical Education, Government of Tripura, 799001, Agartala, Tripura India; 3 Nil Ratan Sarkar Medical College and Hospital, Kolkata, India; 4 The West Bengal University of Health Sciences, Kolkata, India

**Keywords:** *Streptococcus pyogenes* (GAS), *emm* typing, *vir* typing, Virulence regulon, Restriction enzyme digestion, Antibiotic sensitivity patterns, Exotoxin gene

## Abstract

**Background:**

Group A Streptococcus strains causing wide variety of diseases, recently became noticeable in eastern India, are not amenable to standard treatment protocol thus enhancing the possibility of disease morbidity by becoming antibiotic resistance.

**Methods:**

The association of Lancefield group A Streptococcal variation with degree of *vir* architectural diversity was evaluated using *emm* typing and restriction fragment length polymorphism analyses. The antibiotic sensitivity patterns were examined by modified Kirby-Bauer method of disk diffusion. Percentage calculations, 95% confidence interval and one-way ANOVA were used to assess differences in proportions.

**Results:**

Our observations revealed 20 different *emm* types and 13 different *HaeIII vir* typing patterns. A 1.2 kb fragment was found in all *HaeIII* typing pattern. Fragments of 1.2 kb and 550 bp were conserved in majority of the isolates. *HinfI* digestion was found proficient in differentiating the strains of same *vir* typing patterns. Strong predominance of *speC* (85%) and *speF* (80%) genes have been observed encoding exotoxins production. 4 isolates were found to be erythromycin resistant and were of genotype *emm*49. High degree of tetracycline resistance was shown by 53.57% isolates which belonged to 12 different *emm* genotypes.

**Conclusions:**

These findings suggested that in addition to *emm* typing, sequential application of *HaeIII* and *HinfI* restriction enzymes in *vir* typing analysis is an effective tool for group A streptococcal molecular characterization associated with antibiotic resistance.

**Electronic supplementary material:**

The online version of this article (doi:10.1186/s12879-016-2079-9) contains supplementary material, which is available to authorized users.

## Background

*Streptococcus pyogenes* or Lancefield group A *Streptococcus* (GAS) is responsible for a diverse range of clinical manifestations. Acute infection causes pharyngitis, impetigo, scarlet fever, cellulitis, acute bacterial endocarditis and necrotizing fasciitis. Ineffective or delayed treatment, and infection with drug-resistant bacteria eventuates in chronic immune-mediated disorders such as acute rheumatic fever (ARF) and acute glomerulonephritis (AGN) with attended morbidity and mortality worldwide [1–6].

The fast emergence of antimicrobial resistance amongst *S. pyogenes* therefore needs molecular characterization of the newly variant strains. The conventional M serotyping is cumbersome, less effective and demand preparatory requirements [7, 8]. A large number of *S. pyogenes* isolates remains M untypable by the available present range of high titre antisera [9–11]. Alternative techniques used for *emm* and *vir* typing are favored. *emm* typing is based on sequence analysis of *emm* gene [8], that encodes for the major virulence factor M and M-like proteins while *vir* typing is based on amplification of virulence regulon-specific polymerase chain reaction (PCR) amplicon followed by restriction fragment length polymorphism (RFLP) [12].

Different reports have shown a fair prevalence of GAS infections among children [6, 13, 14] and strain variations among the GAS isolates [15, 16]. A sudden world-wide increase in antimicrobial resistance in *S. pyogenes* has been observed during the last few years [17, 18]. Macrolide resistance among GAS has been widespread [19, 20] possibly linking to the spread of a particular clone by selection pressure due to random usage of macrolides [21]. Recent reports on tetracycline resistance identify the ineffectiveness of this drug against infections with GAS [22–26]. Streptococcal pyrogenic exotoxins act as super antigens and stimulate lymphocyte activation to induce fever and extensive proliferation with the consequent massive release of cytokines [27]. Fibronectin binding proteins are considered as major Streptococcal adhesins and amongst them fbp54 is expressed in human host environment [28].

Reports on Streptococcal pyrogenic exotoxin genes and fibronectin binding protein gene expression in noninvasive infections are relatively few from India. The present study tries to establish the correlation of the GAS genotypic variation with the changes in molecular patterns of virulence regulon, antibiotic resistance and expression of pyrogenic exotoxins and fibronectin binding protein genes.

The isolates have been characterized with *HaeIII*, *HinfI* and *XhoI* restriction fragment length polymorphism to evaluate their applicability in *vir* typing analysis following methodology by Gardiner et al. [9, 12].

## Methods

### Isolate selection

The patients presenting with sore throat, different skin lesions and on clinical examination found to be suffering from pharyngitis and/or tonsillitis and pyoderma were selected for study. Samples were collected before administering any antibiotic therapy.

### Patient recruitment

Study patient samples belonged to four age-groups:

Group I 1 month to 3 years [infants]
Group II 4 years to 15 years [school-aged children]
Group III 16 years to 45 years [adults]
Group IV >45 years [middle as well as old age groups]

Detailed clinical history, which includes types of throat infections and skin infections, the period of suffering, was recorded. Age, sex, home address and other information of the patients were recorded. *S. pyogenes* was isolated from the samples at the Department of Biochemistry of Dr. B C Roy Post Graduate Institute of Basic Medical Education and Research (IPGME&R), Kolkata.

### Collection of samples from sore throat patients

Samples for throat culture were obtained by swabbing the patient’s posterior part of upper respiratory tract with a sterile cotton swab.

### Collection of samples from patients with skin infections

Pus samples were collected using a sterile swab stick from ulcerative lesion of pyoderma infections. In case of unopened abscess pus was collected by aspiration.

We got very insignificant number of *S. pyogenes* isolates (two isolates) from throat samples. Therefore we have summarized and interpreted our observations in respect to non-sever infections which include sore throats and pyoderma/ impetigo. It has been done to avoid any bias that might occur during interpretation of the results.

### Sample isolation and storage

Samples were inoculated in 5% sheep blood agar and incubated at 37 °C for 24–48 h. β- hemolytic colonies were selected and screened by Gram staining, bacitracin susceptibility test, catalase test, and finally confirmed as Group A *Streptococcus* by Latex agglutination test using a Streptex kit (Remel, UK). Isolates were stored at −80 °C in Brain Heart Infusion Broth containing 30% glycerol.

### *emm* typing analysis

*emm* typing is based on sequence analysis of *emm* gene encoding for serotype specific M-protein. Genomic DNA was prepared by phenol-chloroform method [29] from bacteria obtained by touching the tips of β-hemolytic single colonies, obtained by subculture from overnight broth culture (BHI broth, Hi-media, Mumbai, India), to avoid contamination in growth in liquid medium. Bacterial cells were treated with mutanolysin and lysozyme solution for 1 h at 37 °C. For *emm* typing analysis, gene was amplified by “all M” primers [15, 30], using PTC150 (MiniCycler^TM^, MJ Research) thermal cycler. PCR amplicon of about 1.4 kb was then sequenced with the forward primer (5′ ATAAGGAGCATAAAAATGGCT 3′). Sequencing was done commercially (Chromous Biotech Pvt. Ltd., India). The sequence was subjected to homology search by Blast search analysis (http://www.cdc.gov/ncidod/biotech/strep/ strepblast.htm). Pair-wise comparison of the nucleotide identities of the first 180 to 200 bases of the N-terminal hypervariable region was conducted and strains which showed ≥ 95% homology with the reference strains were assigned the particular parental *emm* type [15].

### *vir* typing analysis

*vir* typing is based on amplification of virulence regulon that encode for different GroupA Streptococcal virulent proteins. Amplification of *vir* regulon specific PCR amplicon was performed by VUF and SBR primers, as described by Gardiner et al. [12], with few modifications. PCR amplification was carried out in a 50 μl reaction mixture containing 5 μl of template DNA, 1U of TaKaRa LA Taq polymerase, LA PCR Buffer II, 2 mM MgCl_2_, 200 μM of each of dATP, dGTP, dCTP and dTTP, 1 μl of each 20 μM primers. Cycling conditions were modified to include a final extension at 72 °C for 7 min. Then 5 μl of PCR product was electrophoresed on 0.8% agarose gel to check the quality as well as the quantity of the amplicon. RFLP was carried out in 30 μl reaction containing 0.5 μg (10-15 μl) PCR product, RE buffer, 2U of *HaeIII / HinfI / XhoI* restriction enzyme from NEB [9, 12]. Digestion was carried out for 1 h 30 min. Digests were electrophoresed on 1% agarose gel and band patterns were analyzed by visual comparison.

### Identification of toxin and fibronectin-binding protein genes

Sequences specific for *speA*, *speB*, *speC*, *speF* and *fbp-54* were detected with the primers documented by earlier workers [31]. The primer sequences, annealing temperature and amplicon sizes are given in Table 1. Amplification of all genes was carried out under the following conditions: an initial 5 min denaturation step at 96 °C, followed by 30 cycles of denaturation at 96 °C for 55 s, 65 s of annealing at the appropriate temperature for each gene (Table 1), and 70 s of extension at 72 °C, with a final extension step at 72 °C for 5 min. All PCR products were electrophoresed on 1.5% agarose gels. φX174 DNA ladder (GeNei^TM^) and 100 bp Gene ruler (Fermentus) were used as molecular marker.

**Table 1:** Sequences of the PCR primers

Target gene	Primer sequence (5′-3′)	Annealing temp (°C)	Amplicon size (bp)	Reference
*speA*	FW-5΄-TAA GAA CCA AGA GAT GG-3΄ RV-5΄-ATT CTT GAG CAG TTA CC-3΄	44	248	Vlaminckx et al., [31]
*speB*	FW-5΄-AAG AAG CAA AAG ATA GC-3΄ RV-5΄-TGG TAG AAG TTA CGT CC-3΄	42	955	
*spec*	FW-5΄- GAT TTC TAC TTA TTT CAC C-3΄ RV-5΄-AAA TAT CTG ATC TAG TCC C-3΄	42	584	
*speF*	FW-5΄- TAC TTG GAT CAA GAC G-3΄ RV-5΄-GTA ATT AAT GGT GTA GCC-3΄	42	782	
*fbp-54*	FW-5΄-CTT CAG AAT CTG TTT CTT TG-3΄ RV-5΄-AGT TCA CAG GTT GTC TAT TG-3΄	48	595	

### Determination of antibiotic susceptibility

The antibiotic sensitivity of all GAS isolates were tested by the modified Kirby-Bauer method, matching inoculum turbidity to McFarland 0.5 [32], on Mueller-Hinton agar with 5% sheep blood using penicillinG (10unit), cefotaxime (30 μg), erythromycin (15 μg), vancomycin (30 μg), tetracycline (30 μg) and clindamycin (2 μg) [HiMedia, India] disks. Results were interpreted according to Clinical and Laboratory Standards Institute (CLSI) guidelines provided by the CLSI, 2012, as per manufacturer’s instructions [33]. *Streptococcus pyogenes* ATCC19615 strain was used as control.

### Statistical analysis

Majority of counts have been summarized by percentages, key proportion has been expressed within 95% confidence interval. Statistical analysis was done using one-way ANOVA. P value with *P* < 0.05 was considered significant, a difference with *P* < 0.0001 was considered highly significant.

## Results

### Isolation of *Streptococcus pyogenes* and *emm* genotype diversity

Samples were collected from tertiary referral health care hospitals in Kolkata. As these are the most important referral Government hospitals, a large number of patients from different community attend these hospitals. Prior informed consent and clearance from institutional ethical committee were obtained. Samples were collected from patients attending skin and E.N.T OPD of Seth Sukhlal Karnani Memorial Hospital (SSKM) hospital and Calcutta National Medical College & Hospital, from 2009 to December, 2011.

**Fig. 1:**
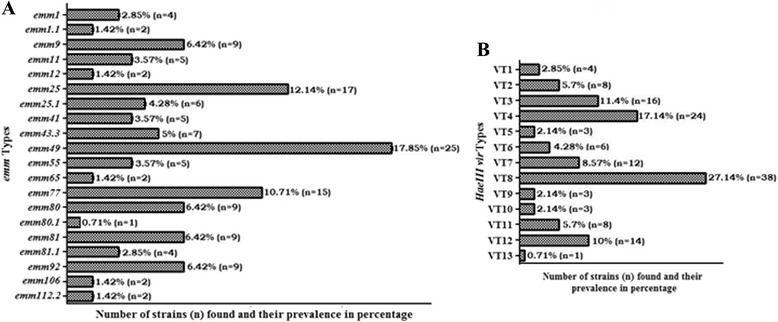
Distribution of different *emm* types (**a**) and HaeIII vir types (**b**) among isolates. 140 isolates yielded interpretable *emm* types and *HaeIII vir* types. Genomic DNA was prepared using the phenol-chloroform method*. emm* gene was amplified by “all M” primers and further sequenced with “all M” forward primer. The sequences were subjected to homology search by Blast search analysis (http://www.cdc.gov/ncidod/biotech/strep/ strepblast.htm) as described in Methods. GAS isolates represented 20 different *emm* types. The most predominant *emm* types found were *emm*49 (17.85%), followed by *emm*25 (12.14%), *emm*77 (10.71%). *emm*9, *emm*80, *emm*92 and *emm*81 was observed in 6.42%. Rests were found in very low percentage (**a**). *vir* regulon specific amplicon was amplified by VUF and SBR primers and resolved on 0.8% agarose gel. RFLP was further carried out using *HaeIII* restriction enzyme and products were resolved on 1% agarose gel. Out of 13 *HaeIII vir* types, the most commonly found *vir* type was VT8 (27.14%), followed by VT4 (17.14%), VT3 (11.4%), VT12 (10%) and VT7 (8.57%) (**b**)

Detailed analysis of isolates from 270 cases identified 140 (51.8%) as GAS expressing interpretable *emm* types. These isolates represented 20 different *emm* types, indicating 14.28% heterogeneity. The sizes of the amplicon were around 1.4 kb. The most common *emm* types among the obtained *emm* types was *emm*49 (Additional file 1) (17.85%), followed by *emm*25 (Additional file 2) (12.14%), *emm*77 (10.71%). The observed frequency for each of *emm*9, *emm*80, *emm*92 and *emm*81 was 6.42%. Others were found in very low percentages (Fig. 1a).

**Fig. 2:**
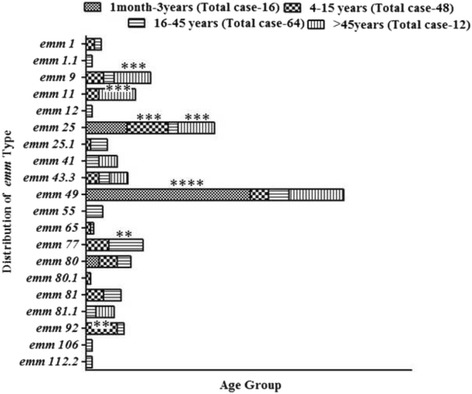
Distribution of different emm types among age groups. Patient samples were categorized in four age-groups as mentioned in Methods. Genomic DNA was prepared using the phenol-chloroform method*. emm* gene was then amplified by “all M” primers and further sequenced with “all M” forward primer. The sequence obtained was then subjected to homology search by Blast search analysis (http://www.cdc.gov/ncidod/biotech/strep/ strepblast.htm). Distribution of different *emm* types among age groups was expressed in percentages as described in Methods. *emm* types of group A streptococci isolated from different age groups revealed that infants (1 month–3 years) are frequently infected by *emm*49 (75%), school-going children (group II) are primarily affected by *emm*25 (18.7%) and *emm*92 (14.5%). A few *emm* strains were detected in adults (group III, 15.6% of *emm*77) and middle or old aged patients (group IV 16.6% of *emm*9, *emm*25 and *emm*11 each). Asterisks indicate a statistically significant correlations (^****^ *P* < 0.0001, ^***^ *P* = 0.0004 and ^**^ *P* = 0.001, 0.008)

Regarding correlations of *emm* typing with age groups, our observations revealed that infants (1 month–3 years) were frequently infected by *emm*49 (75%; *P* value <0.0001), whereas 18.7% school aged children (group II) were affected by *emm*25 (*P* value =0.0004), followed by *emm*92 genotype (14.5%; *P* value =0.001). A few *emm* strains were found in adults belonging to group III (15.6% of *emm*77; *P* value =0.008), and middle or old aged patients in group IV (16.6% of *emm*9, *emm*25 and *emm*11 each; *P* value =0.0004) (Fig. 2).

### *vir* typing patterns diversity

**Fig. 3:**
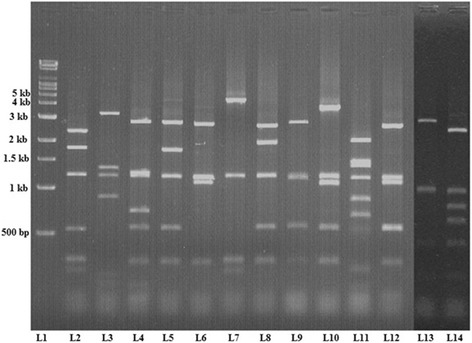
1% Agarose gel containing RFLP profile (vir type) of HaeIII digested PCR products. Genomic DNA was prepared using the phenol-chloroform method*. vir* regulon specific amplicon was amplified by VUF and SBR primers and resolved on 0.8% agarose gel. RFLP was further carried out as described in Methods using *HaeIII* restriction enzyme and products were resolved on 1% agarose gel. Lane 1 = 10 kb Super mix marker; Lanes 2-14 = VT1- VT13

In the present study, 13 different *vir* types among 140 isolates (9.2%) were observed. *vir* types had been identified by RFLP with *HaeIII* restriction enzyme. All isolates were typeable with *HaeIII* enzyme, showing 3–7 bands ranging from 200 bp to 4 kb. The isolates showed a common band at 1.2 kb, and majority of isolates shared fragments at around 200 bp and 550 bp (Fig. 3). Out of 13 different *vir* types, the most commonly found *vir* type was VT8 (27.14%), followed by VT4 (17.14%), VT3 (11.4%), VT12 (10%) and VT7 (8.57%). In our study VT1 represented the reference strain type of ATCC 19615 (Fig. 1b).

On comparing *emm* genotype with *HaeIII vir* typing patterns, it was found that *emm*49, *emm*25, *emm*80, *emm*12 matched with co-existence of VT8, VT3, VT1 and VT4 respectively. In contrast, VT8 was also associated with *emm*65, *emm*11, *emm*77, and VT12 with *emm*92 and *emm*9. VT3 pattern was observed both in *emm*25 type and *emm*25.1 subtype. On the other hand, *emm*81/ *emm*81.1 were noted with VT11 and VT9 respectively, and *emm*1/ *emm*1.1 with VT6, VT10 respectively (Table 2).

**Table 2:** Comparison of *emm* typing/*HaeIII vir* typing/ *HinfI vir* typing of GAS isolates

*emm* type	Corresponding *HaeIII vir* types	Corresponding *HinfI vir* types
*emm*1	VT6	VT_1_
*emm*1.1	VT10	VT_14_
*emm*9	VT12	VT_4_
*emm*11	VT8	VT_8_
*emm*12	VT4	VT_2_, VT_6_,VT_7_, VT_18_
*emm*25	VT3	VT_5_
*emm*25.1	VT3	VT_11_
*emm*41	VT6	VT_17_
*emm*43.3	VT7	VT_18_
*emm*49	VT8	VT_3_,VT_4_
*emm*55	VT2	VT_9_,VT_20_
*emm*65	VT8	VT_16_
*emm*77	VT8	VT_3_,VT_12_
*emm*80	VT1	VT_15_, VT_19_
*emm*80.1	VT5	VT_10_
*emm*81	VT11	VT_21_
*emm*81.1	VT9	VT_13_
*emm*92	VT12	VT_4_
*emm*106	VT8	VT_4_
*emm*112.2	VT13	VT_4_

**Fig. 4:**
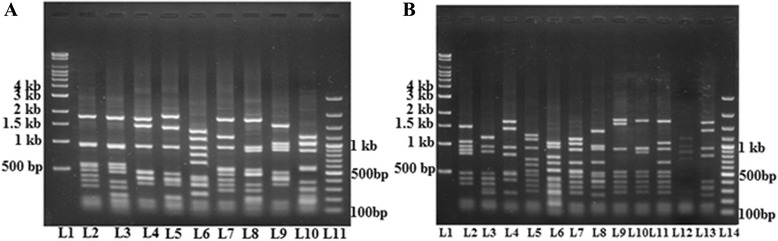
1% Agarose gel containing RFLP profile (vir type) of HinfI digested PCR products. Genomic DNA was prepared using the phenol-chloroform method*. vir* regulon specific amplicon was amplified by VUF and SBR primers and resolved on 0.8% agarose gel. RFLP was performed using *HinfI* restriction enzyme and the products were resolved on 1% agarose gel. Lane 1 = 10 kb Super mix marker; Lanes 2-10 = VT_1_ - VT_9_ ; Lane11 = 100 bp marker (**a**). Lane 1 = 10 kb Super mix marker; Lanes 2-13 = VT_10_ -VT_21_; Lane 14 = 100 bp marker (**b**)

The second restriction enzyme *HinfI*, documented 21 different *vir* typing patterns (VT_1_-VT_21_). *HinfI* digestion was found to generate 6–10 bands, which were around 250 bp to 2 kb (Fig. 4a & b). *HinfI* was found to generate same or different typing patterns for the isolates those producing identical *HaeIII* typing patterns. In comparing the typing patterns of *HaeIII* and *HinfI* digestions, *HinfI* enzyme was found to discriminate VT8 *HaeIII* digestion type into five more subtypes (VT_3_, VT_4_, VT_8_, VT_12_, and VT_16_), VT4 *HaeIII* type into another four more subtypes (VT_2_, VT_6_, VT_7_, VT_18_) and each of VT1, VT2, VT3 into two more subtypes (Table 2).

**Fig. 5:**
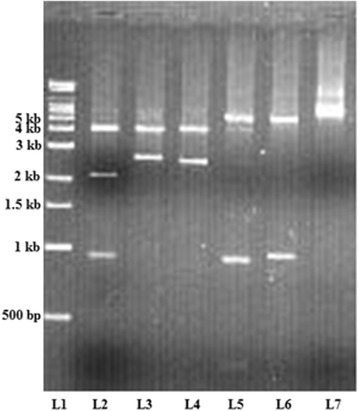
1% Agarose gel containing RFLP profile (vir type) of XhoI digested PCR products. Genomic DNA was prepared using the phenol-chloroform method*. vir* regulon specific amplicon was amplified by VUF and SBR primers and resolved on 0.8% agarose gel. RFLP was performed using *XhoI* restriction enzyme and the products were resolved on 1% agarose gel. Lane 1 = 10 kb Super mix marker; Lanes 2-7 = VTi- VTvi

In our study, the third restriction analysis by digestion with *XhoI* recognized 6 different typing patterns (VTi-VTvi), with fragments around 1 to 6 kb among 140 isolates (Fig. 5). Study showed the most frequent VT patterns by *XhoI* digestion were 34.28, 14.99, and 12.85% for VTv, VTiii and VTiv respectively. VTvi, showing only a minor band, was found in 24.99% isolates.

### Identification of exotoxin and fibronectin-binding protein genes

Study highlighted that only 6 (4.2%) isolates was found to contain *speA* gene, whereas majority of isolates contained *speC* (85%) and *speF* (80%) genes. The rate of occurrence of *speB* and *fbp-54* genes was 58 and 30%, respectively.

### Evaluation of antibiotic susceptibility patterns of *S. pyogenes*

In the present study, isolates were found highly sensitive (76.42%) to penicillin with 23.57% of intermediate susceptibility. Erythromycin sensitivity was very high (88.57%), and only 4 (2.85%) found resistant (95% CI-0.09%–05.61). All isolates were susceptible to vancomycin and clindamycin. Cefotaxime sensitivity was very high (91.42%) with only 8.57% of isolates showing intermediate sensitivity. By contrast, resistance (53.57%) and intermediate resistance (21.42%) to tetracycline were high (Table 3).

**Table 3:** Antibiotic susceptibility patterns of *S. pyogenes*

Sl No.	Antimicrobial agent (amount/disk)	Patterns of susceptibility (in percentage) with respect to diameter of zone of inhibition in mm		
		Resistant	Intermediate	Sensitive
1.	PenicillinG (10unit)	-	33 (23.57%)	107 (76.42%)
2.	Erythromycin (15mcg)	4 (2.85%)	12 (8.57%)	124 (88.57%)
3.	Cefotaxime (30mcg)	-	12 (8.57%)	128 (91.42%)
4.	Vancomycin (30mcg)	-	-	140 (100%)
5.	Tetracycline (30mcg)	75 (53.57%)	30 (21.42%)	35 (24.99%)
6.	Clindamycin (2mcg)	-	-	140 (100%)

### Correlation of antibiotic susceptibility patterns with emm typing

Relation between *emm* types and antibiotic sensitivity patterns was evaluated for studying their correlations. Strain *emm*43.3 and *emm*9 were found to be sensitive to all six antibiotics. Three children and one adult showing erythromycin resistance cases were of genotype *emm*49. Out of 20 *emm* types documented, 12 *emm* types were found responsible for tetracycline-resistance (Table 4).

**Table 4:** Association between *emm* types and antibiotic sensitivity patterns

Sl No.	Antimicrobial agent	Susceptibility of *emm* types		
		Sensitive	Intermediate	Resistant
1.	PenicillinG	*emm-*25, 25.1, 49, 80, 9, 11, 81, 81.1, 43.3, 106, 41, 92, 77, 55, 1	*emm-*112.2, 25, 49, 81, 1.1, 65, 80.1, 12	-
2.	Cefotaxime	*emm-*25, 25.1, 49, 80, 80.1, 9, 11, 81, 81.1, 106, 41, 43.3, 92, 77, 55, 1, 1.1	*emm-*25, 49, 81, 12, 112.2, 65	-
3.	Erythromycin	*emm-*43.3, 25, 25.1, 49, 80, 80.1, 9, 11, 12, 81, 81.1, 106, 41, 92, 77, 55, 1, 1.1	*emm-*49, 112.2, 65, 1	*emm-*49
4.	Clindamycin	*emm-*25, 25.1, 49, 43.3, 80, 80.1, 1, 1.1, 9, 11, 12, 81, 81.1, 106, 41, 92, 77, 55, 112.2, 65	-	-
5.	Tetracycline	*emm-*25, 43.3,49, 9, 77, 80.1, 81, 65	*emm-*25.1, 80, 49, 81, 81.1, 12	*emm-*1, 1.1, 41, 55, 81, 81.1, 77, 49, 11, 106, 112.2, 92
6.	Vancomycin	*emm-*25, 25.1, 49, 43.3, 80, 80.1, 1, 1.1, 9, 11, 12, 81, 81.1, 106, 41, 92, 77, 55, 112.2, 65	-	-

## Discussion

In the present study, the *emm* and *vir* type diversity among GAS isolates obtained from different patients was analyzed. Reports by earlier workers documented 57.5, 19.71 and 40% heterogeneity with *emm*49, *emm*77 and *emm*74 genotypes to be the most prevalent types respectively [15, 34, 35]. In another study, 32.35% heterogeneity was reported [16]. A Norweigian study revealed 44% variation with *emm*1 to be the most prevalent genotype [36]. In a recent outbreak of GAS infections, the most uncommonly reported *emm* type 58 was shown to be most frequent isolate [37].

In present study, *emm* typing resulted in 14.28% heterogeneity in the isolated GAS strains which is indicative of lower strain variation in this context. The most detectable type found was *emm*49 (17.85%). Thus different values of heterogeneity in the isolates were observed by the present and earlier investigators, might be due to variation of prevalent strains in different geographical regions having different climatic conditions. It could possibly be predicted that the genetic heterogeneity could be temperature and humidity dependent, however this fact requires a complete different investigation. The results of this study demonstrated some *emm* types and their subtypes, like *emm*81/*emm*81.1, *emm*25/*emm*25.1 and *emm*1/*emm*1.1, emerged due to point mutation, reflecting in the origin of N-terminal *emm* sequence variability and implying the importance of *emm* strain typing (sequencing data not shown).

As children are more susceptible to GAS infections [38–40], and as associated *emm*-types and clinical presentations are influenced by population immunity and strain tropism [41], the *emm* distribution patterns in relation to patient’s age, the children in particular, are significant. In this study occurrence of *S. pyogenes* infection was found average. As children are more susceptible to GAS infections with attendant life-threatening remote sequelas (RHD and AGN) as well, it is believed that the dominance of *emm*49 as well as other frequently occurring *emm* types in infant and children needs an independent and systematic study on association of immunological parameters with *emm* types.

*Vir* typing measures the changes in the region of the genome that determines major virulence factors that are involved in anti-phagocytic activity, attachment, colonization and in avoiding recognition by the immune systems [12]. The *HaeIII vir* typing patterns in the present study were found dissimilar to other reported typing patterns. So we assigned our own VTs to analyze the typing patterns of the isolates from eastern region of India. We screened 13 *vir* types from 140 isolates by *HaeIII* digestion, which accounted 9.28% diversity in GAS isolates. Here we observed that *vir* types were entirely different from the reported typing patterns of Australia, Romania and northern India. *HaeIII vir* typing patterns of Australian GAS isolates produced 4 to 8 fragments ranging from 200 bp to 4 kb, where 1.4 kb, 500 bp, and 275 bp were found common in the isolates tested [9]. *vir* typing patterns of Romania yielded 2 to 8 fragments ranging from 100 bp to 4.2 kb with a common fragment at 1.2 kb [42]. Reports from north India showed 4 to 6 fragments varying from 300 bp to 5 kb where 300 bp, 550 bp and 1.2 kb were common for the majority of isolates [15]. We found 3 to 7 fragments of 200 bp to 4 kb and a 1.2 kb fragment was found common to all isolates. The majority of isolates shared the fragments of around 200 bp (81.42%) and 550 bp (87.85%). On comparing our present findings with those previous observations, a 1.2 kb fragment was found common in most of the study regions (except Australian typing patterns). In India, in addition to 1.2 kb, a 550 bp was also found common to the majority of isolates. Thus, these two segments of the virulence regulon of *S. pyogenes* remain conserved in Indian strain types.

The overlaps between *emm* and *vir* typing showed that one specific *emm* type was always represented by one particular *vir* type whereas one *vir* type was shared by more than one *emm* types. However, in two cases, different VTs were obtained for isolates of one *emm* type and its subtype. This finding supports the concept that the heterogeneity demonstrated by *vir* typing is primarily due to variations among *emm* genes rather than diversity in the architecture of the *vir* regulon [9] and thus, an insertion or deletion of one or more nucleotide not only categorizes a specific *emm* type into its subtypes but also changes the expression of its virulence regulon. Thus *vir* typing analysis is found more informative than *emm* typing, regarding architectural variation of virulence regulon conferring the virulence on *S. pyogenes.*

Furthermore the use of the second restriction enzyme *HinfI* appeared to be very useful in discriminating *HaeIII* typed isolates, which supports the concept of Gardiner et al. [9]. In the present study, though *HinfI* was found proficient in distinguishing the isolates of the same *HaeIII vir* types and also generate more fragments comparing *HaeIII* but it did not generate any conserved fragments, unlike *HaeIII* digestion. Therefore, successive use of these two enzymes can be a tool for *vir* typing analysis. *XhoI* digestion produced fewer fragments and was less informative.

In order to study the strain characteristics contributing to virulence patterns, the isolates were evaluated for the presence of different toxin and fibronectin binding protein genes. Extracellular proteins that reportedly play a role in the pathogenesis of GAS are represented by streptococcal exotoxins, a few of which have superantigenic properties that allow them to activate large subsets of T cells, with massive cytokine release as a consequence [31]. Previous report from India showed lower incidence of *speA* gene (5.8%) and higher incidence of *speB and speF* gene (92.3, 86.5%) in noninvasive infection [43]. In contrast, the current study showed *speA* gene less contributing for GAS infections, whereas the observed predominant occurrence of *speC* and *speF* genes strongly suggest their association with incidence of infections. Association of *SpeB* and *fbp-54* genes was noticed in less number of cases and probably partly contributes towards virulence. It is of considerable interest to study the correlation of these exotoxin gene expressions with cytokines activation in this region by further study.

Another important aspect of this study is GAS antibiotic sensitivity patterns and their correlation with *emm* genotyping. Penicillin has been used routinely for treating GAS infections for over 50 years, and yet GAS has remained penicillin susceptible [44, 45]. Since penicillin may be given at higher doses without ill effects, the observed intermediate sensitivity in our study may have less impact. A study from north India [7] showed diminished susceptibility in 20.6% of strains which is in support of our observation but contrast to previous reports of 100% susceptibility [46, 47]. Erythromycin resistance and therapeutic failure have been observed to be on the rise worldwide due to their increased medicament [48–50]. In this study, sensitivity to erythromycin was found very high. The observed erythromycin resistant cases were though small in number, but alarming in this region as these were obtained mostly from children, and erythromycin is the 1^st^ alternative drug of choice for penicillin hypersensitive patients as well. Interestingly, all four erythromycin resistant strains were of genotype *emm*49, suggesting that the genotype’s association with the emerging erythromycin-resistance in this region. Association of erythromycin resistance and *emm* typing has been documented earlier. *emm*12 from Korea and Japan [51], *emm*12 and *emm75* from Pittsburgh [52], *emm*90 from Hawaii [53], *emm* 4, *emm* 28, and *emm* 77 from Western Greece [54] showed strong association with erythromycin resistance. In present study, association is different from earlier workers as *emm*49 was constantly associated with the erythromycin resistance. In spite of clindamycin resistance report [55] earlier, all isolates of present study were found sensitive to clindamycin and vancomycin. High tetracycline resistance among *S. pyogenes* isolates have been reported from different parts of the World [22, 44, 56, 57]. In the present study, high percentage of resistance was shown by tetracycline, which is in corroboration with the previous findings. 14 and 28 different tetracycline-resistant *emm* types were identified in studies from Spain and Tunisia, respectively [58, 59]. Present study identified 75 tetracycline-resistant isolates, belonging to 12 different *emm* genotypes. These findings indicated that for study of different regions, *S. pyogenes* strains are approaching tetracycline-resistance, attributable to modifications in *tet* (M) gene commonly associated with the conjugative transposon Tn916 [59].

## Conclusion

The findings in this study highlight a novel *vir-emm* correlative dual genotyping analysis with implications in the development of a universally effective vaccine and a new diagnostic tool. *speC* and *speB* exotoxins may have important contribution to noninvasive Streptococcal infections. Observations on antibiotic sensitivity patterns emphasized the need for continued monitoring of antibiotic sensitivity and genotyping of *S pyogenes* for reducing the burden of antibiotic resistance.

## Additional files

Additional file 1: sequencing data *emm*25. (TIF 741 kb)
Additional file 2: sequencing data *emm*49. (TIF 690 kb)

## Abbreviations

AGN: Acute glomerulonephritis
ARF: Acute rheumatic fever
BHI broth: Brain heart infusion broth
CLSI: Clinical and Laboratory Standards Institute
E.N.T. OPD: Ear nose and throat outpatient department
GAS: GroupA *Streptococcus*
PCR: Polymerase chain reaction
RFLP: Restriction fragment length polymorphism
RHD: Rheumatic heart disease
*S. pyogenes*: *Streptococcus pyogenes*
