# Supplementary figures and images for: Molecular characterization and evaluation of the emerging antibiotic-resistant Streptococcus pyogenes from eastern India
Source: BMC Infect Dis. 2016 Dec 12;16:753. doi: 10.1186/s12879-016-2079-9 (PMC5153692; doi:10.1186/s12879-016-2079-9)

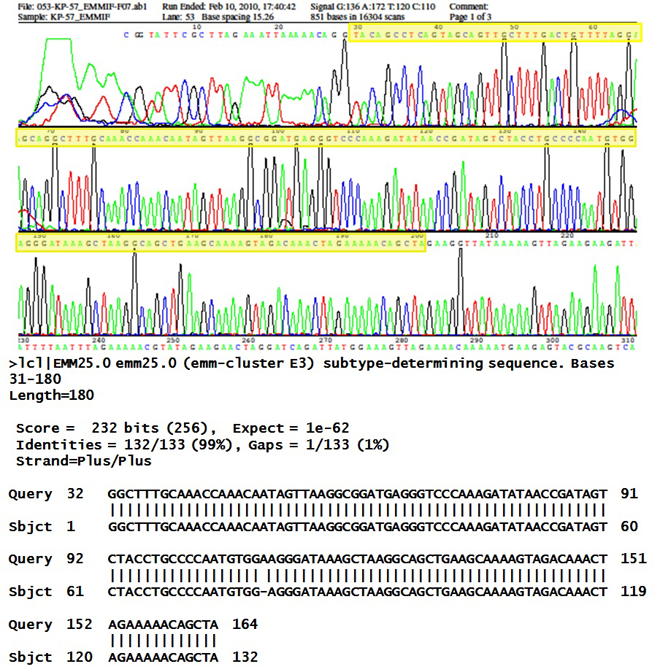

Supplement: Additional file 1: — sequencing data emm25. (TIF 741 kb) [file 12879_2016_2079_MOESM1_ESM.tif]

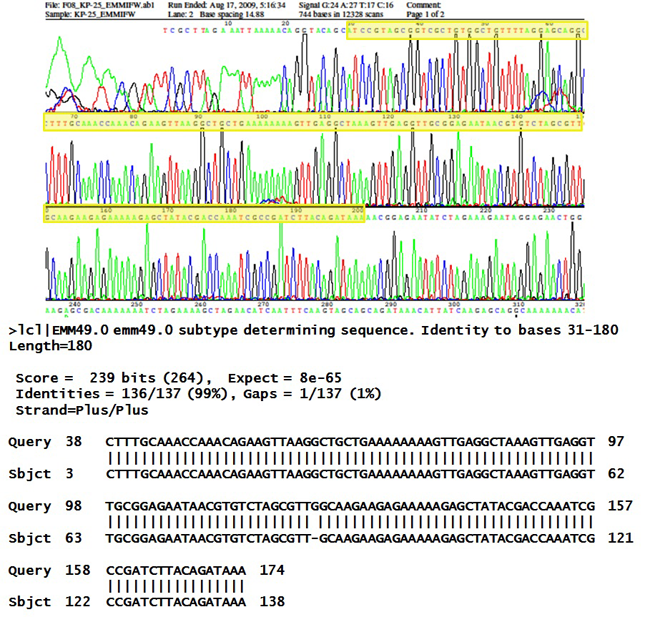

Supplement: Additional file 2: — sequencing data emm49. (TIF 690 kb) [file 12879_2016_2079_MOESM2_ESM.tif]
